# PS Integrins and Laminins: Key Regulators of Cell Migration during *Drosophila* Embryogenesis

**DOI:** 10.1371/journal.pone.0023893

**Published:** 2011-09-16

**Authors:** Jose M. Urbano, Paloma Domínguez-Giménez, Beatriz Estrada, María D. Martín-Bermudo

**Affiliations:** 1 Centro Andaluz de Biología del Desarrollo, (CSIC)-Universidad Pablo de Olavide, Sevilla, Spain; 2 Centro Andaluz de Biología Molecular y Medicina Regenerativa, (CSIC)-Universidad de Sevilla, Sevilla, Spain; Institute for Research in Biomedicine, Spain

## Abstract

During embryonic development, there are numerous cases where organ or tissue formation depends upon the migration of primordial cells. In the *Drosophila* embryo, the visceral mesoderm (vm) acts as a substrate for the migration of several cell populations of epithelial origin, including the endoderm, the trachea and the salivary glands. These migratory processes require both integrins and laminins. The current model is that αPS1βPS (PS1) and/or αPS3βPS (PS3) integrins are required in migrating cells, whereas αPS2βPS (PS2) integrin is required in the vm, where it performs an as yet unidentified function. Here, we show that PS1 integrins are also required for the migration over the vm of cells of mesodermal origin, the caudal visceral mesoderm (CVM). These results support a model in which PS1 might have evolved to acquire the migratory function of integrins, irrespective of the origin of the tissue. This integrin function is highly specific and its specificity resides mainly in the extracellular domain. In addition, we have identified the Laminin α1,2 trimer, as the key extracellular matrix (ECM) component regulating CVM migration. Furthermore, we show that, as it is the case in vertebrates, integrins, and specifically PS2, contributes to CVM movement by participating in the correct assembly of the ECM that serves as tracks for migration.

## Introduction

Cell migration plays a key role in a wide variety of biological phenomena. During embryogenesis, many cells travel substantial distances to reach their final destinations, where they aggregate to form tissues. In the adult organism migration remains prominent in both normal physiological conditions, as well as pathological situations. During this process, a migratory cell first breaks the adhesive bonds with their neighboring cells and surrounding matrix. Concomitantly, the cell establishes new dynamic contacts with the substratum over which it will migrate, to serve as traction points that will propel its movement. This behaviour, an intricately-coordinated and controlled processes in normal cells, becomes destructive and damaging when acquired by cancerous cells. Hence a better understanding of the molecular mechanisms that transform stationary cells into migratory cells would not only be useful to gain a deeper insight of organogenesis, but also help to understand, treat or even prevent cancer metastasis.

Among the adhesion receptors found to be involved in the migration of different cell types, integrins constitute a major family of receptors promoting cell migration. Integrins are heterodimeric receptors consisting of α and β chains that are present and conserved in all metazoan animals. However, while in mammals, eight β and eighteen α subunits have been characterized, the *Drosophila* genome encodes two βs (βPS and βν) and five αs (α1–α5) subunits. The role of integrins in cell migration includes both a structural and a signalling aspect. On one hand, they act as links between the ECM and the actin cytoskeleton, allowing cells to grasp to the substratum and move. On the other hand, they modulate signalling components that control cell migration, such as members of the Rho family of GTPases, focal adhesion kinase, Src kinase, and the Erk and JNK pathways. Finally, during development, integrins have diverse ways of contributing to cell migration. They can be required in migrating cells for their movement and/or in the surrounding cells to assemble an ECM substratum for migration [1].

In the *Drosophila* embryo, three tissues of epithelial characteristics are known to require integrin function for their proper migration: the midgut endoderm, the visceral branches of the developing trachea and the salivary glands [2], [3], [4], [5], [6]. These three cell types use the same substratum for their migration, the visceral mesoderm (vm) [4], [6], [7], [8]. For both the trachea and the endoderm, a requirement for PS2 was demonstrated in the vm substrate [3], [4]. In contrast, different integrins are involved on the side of the migrating cells. Thus, PS1 is required in the visceral branches of the trachea [4], while both αPS1βPS and αPS3βPS and, with a limited contribution βν, are required in the endodermal cells [3], [5]. PS1 and PS2 functions during cell migration seem to be distinct and specific, as they cannot substitute for each other [3], [4]. Because PS1 is expressed in epithelial cells whereas PS2 is found in mesodermal cells, the specificity of the functions may arise from the presence of unique downstream effectors in the different cell types. Alternatively, PS1 integrin function in cell migration could be due to its ability to mediate ligand-affinity interactions necessary to promote the migratory function of integrins. If this were the case, one would expect PS1 integrin to mediate migration in different cell types and not only in epithelial cells. We decided to test this by analysing the migration of the caudal visceral mesodermal (CVM) cells, a group of mesodermal cells that also uses the vm as a substratum.

**Figure 1:**
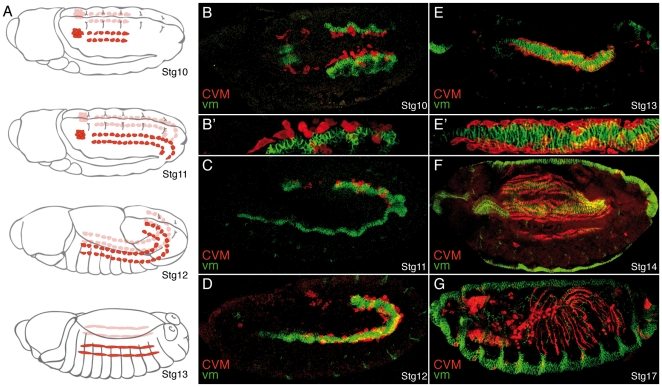
Migration of the caudal visceral mesodermal cells. Caudal visceral mesodermal cells (CVM, red) migrate over the visceral mesoderm (vm, green). (A) Schematic diagrams illustrating the two clusters of migrating CVM cells on each side of the embryo (red) at different embryonic stages (adapted from [69]. (B–G) Lateral view, with the exception of B (dorsal view), of embryos carrying the CVM marker croc-LacZ stained for βGal (red) to label CVM cells and FasIII (green) to label the vm. In all figures, embryos are oriented with anterior to the left. (B) At stage (Stg) 10, CVM cells start their migration as two clusters in close contact with the vm (Magnification in B′). (C, D) During Stgs11 and 12, CVM cells move anteriorly over the vm. (E, E′) CVM cells stop migrating at Stg13 when they reach the foregut-midgut transition. (F) At Stg 14, CVM cells spread regularly over the vm. (G) At Stg17, CVM cells have stretched in anterior-posterior direction and form regularly spaced longitudinal fibers.

CVM cells are the progenitor of the longitudinal muscles, the outer sheet of muscles surrounding the midgut endoderm [9], [10]. Initially, they are located at the posterior tip of the mesodermal germ layer. At the onset of germ band retraction, they split into two bilaterally symmetrical clusters on each side of the embryo and begin to move anteriorly over the visceral mesoderm. During their last phase of migration CVM cells spread regularly over the visceral mesoderm, acquire a spindle shape and form regularly spaced longitudinal fibers (Fig. 1; [9], [10]. In mutant embryos where the vm is abnormal or absent, such as *twist* or *binou* (*bin*) mutants, the CVM cells internalize and arrange in two clusters ventrolaterally but cannot migrate anteriorly [10], [11]. These results have led to the proposal that the vm serves as a substratum for CVM migration. Furthermore, although it has not been formally proven, the current model is that cells do not migrate over the vm directly but rather they might use an unidentified ECM substratum present on the vm [12].

In this work, we show that PS integrins are required for CVM migration. CVM cells lacking the PS integrins constantly form and retract protrusions without efficient movement, suggesting a PS function for the adhesion of protrusions to the substrate. We demonstrate that while PS2 is required in the vm, PS1 is expressed and specifically required in CVM cells. Since these motile cells are of mesodermal origin, our results strongly suggest that PS1 is capable of mediating cell migration irrespective of the origin of the tissue. Furthermore, we show that PS1 function in both mesodermal and epithelial migrating cells is highly specific, as it cannot be replaced by PS2. This specificity resides both in the cytoplasmic and the extracellular domains of the αPS1 subunit. In addition, we show that removal of laminins, and specifically lamininW (α1,2;β1;γ1), inhibits CVM migration, demonstrating that CVM cells use an ECM substratum present on the vm for their migration. Furthermore, we show that PS2 integrins contribute to CVM movement by the assembly of an optimal ECM substrate.

## Results

### Role of integrins during CVM migration

**Figure 2:**
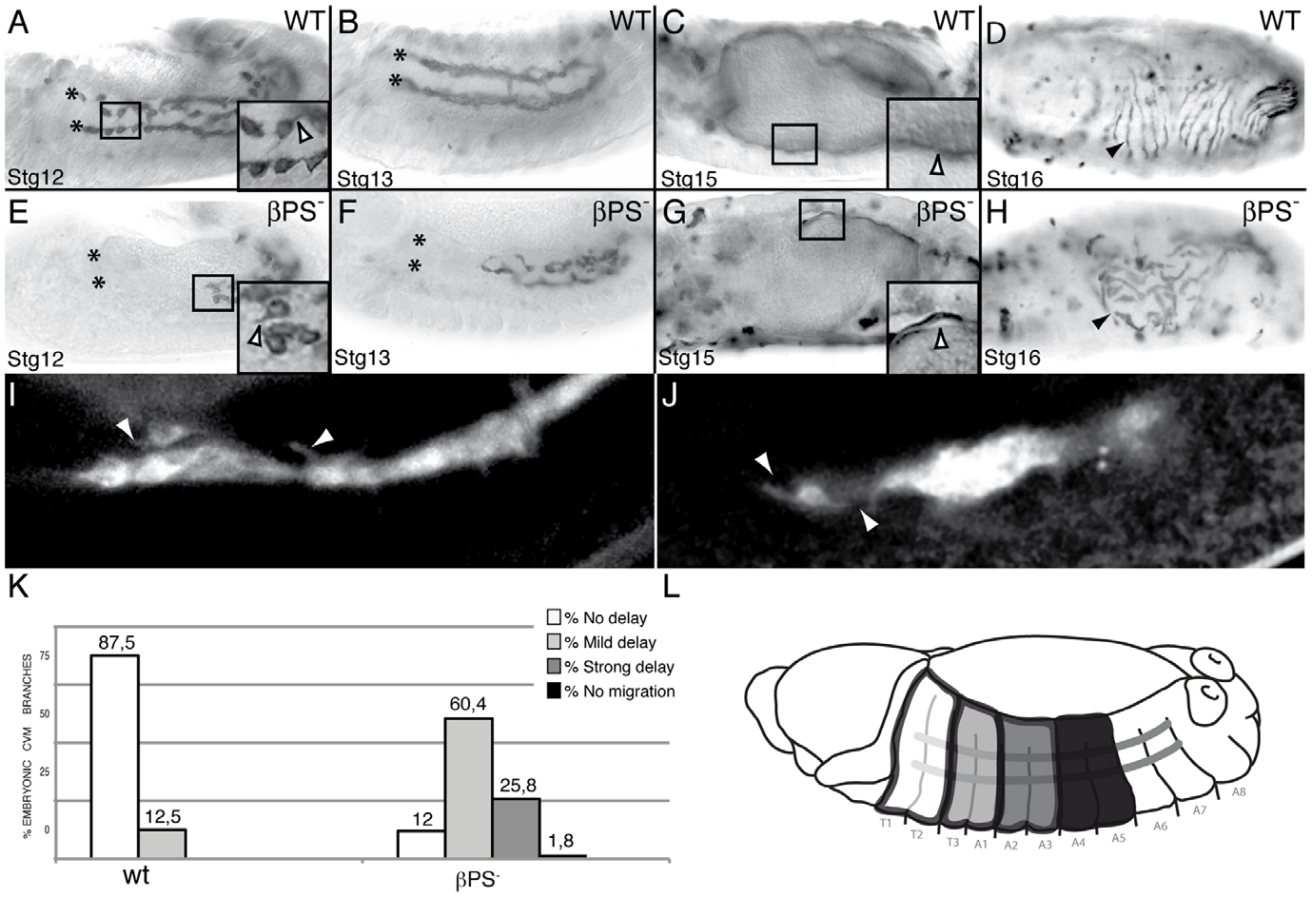
CVM migration is delayed in embryos lacking the βPS subunit. (A–D) Wild type embryos and (E–H) βPS maternal and zygotic mutant embryos. CVM cells are visualized by the expression of the transmembrane protein CD2 driven by the CVM G447.2-GAL4 and detected with an anti-CD2 antibody. (A, B, E, F) During germ band retraction, βPS mutant CVM cells show a delay in their migration although they can still send projections as wild type cells do (arrowhead in magnification in black box). (G, H) At Stgs15 and 16, the longitudinal fibers of βPS mutant embryos detach from the underlying vm (G, arrowhead in magnification in black box) and do not spread properly (arrowhead, H). (I, J) Snap shots from live imaging recording embryos carrying the CVM 5053A-GAL4 driving src-GFP. Both wild type (I) and βPS mutant (J) CVM cells send projections (arrowheads) while migrating. (K) Quantification of the CVM migration phenotype in Stg13 embryos of the indicated genotypes. (L) Schematic diagram of a Stg13 embryo showing the distance reached by CVM cells. In all figures, asterisks mark the foregut-midgut transition, where CVM cells stop migrating.

The *Drosophila* genome contains two integrin β subunits βPS and βν. The βPS integrin, encoded by the gene *myospheroid* (*mys*), is widely expressed while βν is mostly expressed in the midgut endodermal cells [5]. The βPS subunit forms heterodimer with all five αPS subunits encoded by the *Drosophila* genome. Since only αβ heterodimers are transported to the cell surface [13], elimination of the βPS subunit results in a complete absence of all PS integrin function. Thus, in order to study the possible role of PS integrins during CVM migration, we first examined CVM migration in embryos lacking the βPS subunit. Around the onset of germ band retraction, CVM cells split into two bilaterally symmetrical clusters on each side of the embryo and by late stage 11, they begin to move anteriorly over the vm ([9], [10], Movie S1 and Fig. 1). During subsequent stages, the clusters migrate along the dorsal and ventral edge of the midgut in close contact with the vm until, by stage 13, they reach the midgut-foregut transition, where the proventriculus inserts ([9], [10] and Fig. 1 and Fig. 2A, B). Finally, as the midgut encloses the yolk, CVM cells acquire a spindle shape, stretch and form regularly spaced longitudinal oriented fibers, which spread regularly over the underlying circular muscles ([9], [10] and Fig. 1 and Fig. 2C, D). The βPS subunit is maternally deposited in the embryo, thus to completely eliminate PS integrin function, we generated embryos lacking both maternal and zygotic βPS expression (referred to hereafter as βPS^−^ mutant embryos, see material and methods). To visualize the morphology of CVM cells, we have expressed cell membrane markers, such as Src-GFP and CD2, in CVM cells using the GAL4 lines G447.2 or 5053A ([9], [14] and Fig. 2). Quantification of CVM migration over the vm was done by scoring all four CVM branches at stage 13, which is when wild type CVM cells have reached the midgut-foregut transition and have stopped their migration. We used the segmental grooves of the embryo as reference points since we noticed that the midgut-foregut transition is at the level of the boundary between the first (T1) and second (T2) thoracic segments. It is worth mentioning here that the initial steps of CVM migration seem to be independent of the vm. CVM cells can internalize, arrange in two clusters ventrolaterally and disperse over the posterior midgut in embryos lacking the vm, such as *twist* or *biniou* (*bin*) [10], [11]. By analyzing *bin* mutant embryos at stage 13, when germ band retraction is complete, we found that CVM cells become positioned at the level of the fourth abdominal segment, A4 (Figure S1B). In this context, we can use A4 as a landmark for the position that CVM cells reach independently of their movement over the vm. Taking this into account, we used the following criteria to quantify CVM migration over the vm: no migration indicates CVM branches which have stopped at the level of A4, strong delay indicates CVM branches that stay in A3 and A2, mild delay corresponds to CVM branches that stop their migration at the level of A1 and T3 segments and no delay refers to CVM branches that have reached the T1–T2 boundary (Fig. 2L). Using this criterion, we found that 87.5% of wild type CVM branches show no delay and 12.5% show a mild delay (Fig. 2K). In contrast, even though CVM cells can invaginate and split in two clusters in βPS^−^ mutant embryos, 1,8% of CVM branches do not migrate, 25.8% show a strong delay, 60.4% show a mild delay and only 12% migrate as wild type (Fig. 2E, F, K). These results show that while integrins are not required for the initial vm-independent migration of CVM cells, they play an important role during their migration over the vm. Later in development, at stages 15 and 16, integrins are also required for the close apposition of the longitudinal muscles to the vm and for proper spreading of the longitudinal fibers (Fig. 2G, H).

The migration defects observed in βPS^−^ mutant embryos could be attributed to a failure in CVM differentiation. The normal expression in these mutants of reporter genes driven by two independent CVM GAL4 lines, G447.2 or 5053A (see above), argues against this. Nevertheless, we further tested this possibility by analyzing the expression of other CVM markers, such as *bHLH54F-LacZ*, *dorsocross-GFP* (*doc-GFP*), *crocodile-LacZ* (*croc-LacZ*) and the anti-*couch-potato* (*cpo*) antibody [15], [16], in βPS^−^ mutant embryos and found it was not affected (Figure S2). These results show that loss of integrin function does not affect CVM cell fate.

Although βν is mainly expressed in the midgut, low levels might be expressed in other tissues where it could provide compensatory integrin function. To test this possibility we analyzed CVM migration in the absence of βν and found it was not affected (data not shown). Next, we tested whether the function of βν was masked by the presence of βPS by examining CVM migration in embryos lacking zygotic and maternal βPS and βν integrins. We found that removal of βν function did not enhance the strength of the βPS phenotype, demonstrating that βν is not required for CVM migration (data not shown).

Talin, the first integrin-binding protein identified [17], is essential for most βPS integrin functions [18]. However, the role of Talin in cell migration has only been analyzed in the endoderm, where Talin is required for the early βPS-dependent phase of migration [5]. Thus, we have analyzed CVM migration in embryos lacking both maternal and zygotic contributions of Talin, and found that the phenotype was identical to the loss of βPS (Figure S1C, D). This suggests that Talin is required for CVM migration.

Integrins are required at different steps of a migratory process: protrusion formation, adhesion, cell traction, de-adhesion and tail retraction [19]. In two *Drosophila* tissues, the migrating endoderm and the amnioserosa, integrins are required for the formation of protrusions [3], [20]. However, we could detect projections in the CVM cells of fixed βPS^−^ mutant embryos (Fig. 2E). To better analyze the role of integrins in protrusion formation during CVM migration, we analyzed this process *in vivo* using a membrane tagged version of GFP, Src-GFP. Wild type CVM cells sent out protrusions as they migrate anteriorly (Movie S2 and Fig. 2I). We found that βPS^−^ CVM cells could indeed send out protrusions (Movie S3 and Fig. 2J). However, in contrast to wild type, mutant CVM cells went through cycles of extending and retracting protrusions without an efficient movement (Movies S2, S3). These results led us to propose that βPS function is most likely required for the proper adhesion of the protrusions to the substrate and not for the formation of these structures per se.

### αPS1 and αPS2 cooperate to mediate CVM migration

**Figure 3:**
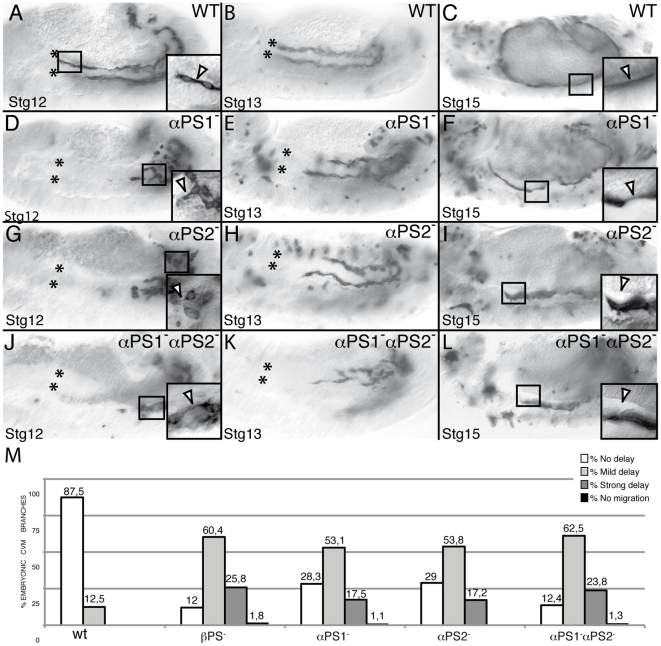
PS1 and PS2 integrins are required for proper CVM migration. CVM cells are visualized using the combination G447.2-GAL4/UAS-CD2 and an anti-CD2 antibody staining. CVM cells from Stgs12 and 13 αPS1 (D, E, M) and αPS2 (G, H, M) mutant embryos are delayed in their migration as compared with wild type cells (A, B, M, yet they still send projections (D, G, arrowhead in magnification in black box) (F, I) In addition, CVM fibers from these mutant embryos detach from the vm at Stg15 (arrowhead in magnification in black box). (J–L) These phenotypes are enhanced in αPS1αPS2 double mutant embryos, phenocopying the defects observed in βPS mutant embryos. (M) Quantification of the CVM migration phenotype in Stg13 embryos of the designated genotypes.

Three out of the five α subunits, αPS1 (encoded by the gene *mew*, [21]), αPS2 (encoded by the gene *if*, [22]) and αPS3 (encoded by the gene *scab*, [23]), have been implicated in different migratory processes during *Drosophila* embryogenesis, such as the migration of the midgut endoderm primordium, the salivary glands and the tracheal visceral branches [2], [3], [4], [5], [6]. In order to test the specific requirements of these α subunits in mediating CVM cell migration, we analysed the phenotype of embryos lacking the different individual αPS subunits. These α subunit genes do not have any detectable maternal contribution [21], [23], [24]. We found that in embryos lacking αPS1 1,1% of CVM branches do not migrate, 17,5% show a strong delay, 53,1% show a mild delay and only 28,3% migrate as wild type (Fig. 3A, B, D, E, and M). Similarly, in αPS2^−^ mutant embryos, 17.2% and 53.8% of the CVM branches show a strong and a mild delay, respectively, and 29% migrate normally (Fig. 3A, B, G, H, M).

In addition, we found that αPS1^−^αPS2^−^ double mutant embryos showed a more severe delay in cell migration than the single mutants alone, phenocopying the migration defects seen in βPS^−^ mutant embryos (Fig. 3K, M). Later, attachment and spreading of fibers is also affected in both single and double mutant embryos (Fig. 3F, I, L). We could not detect any defect in CVM migration in embryos mutant for αPS3 (data not shown). Taken altogether, these results suggest that αPS1 and αPS2 are the α subunits that mediate βPS integrin function during CVM migration.

### αPS1 is expressed in CVM cells during their migration

**Figure 4:**
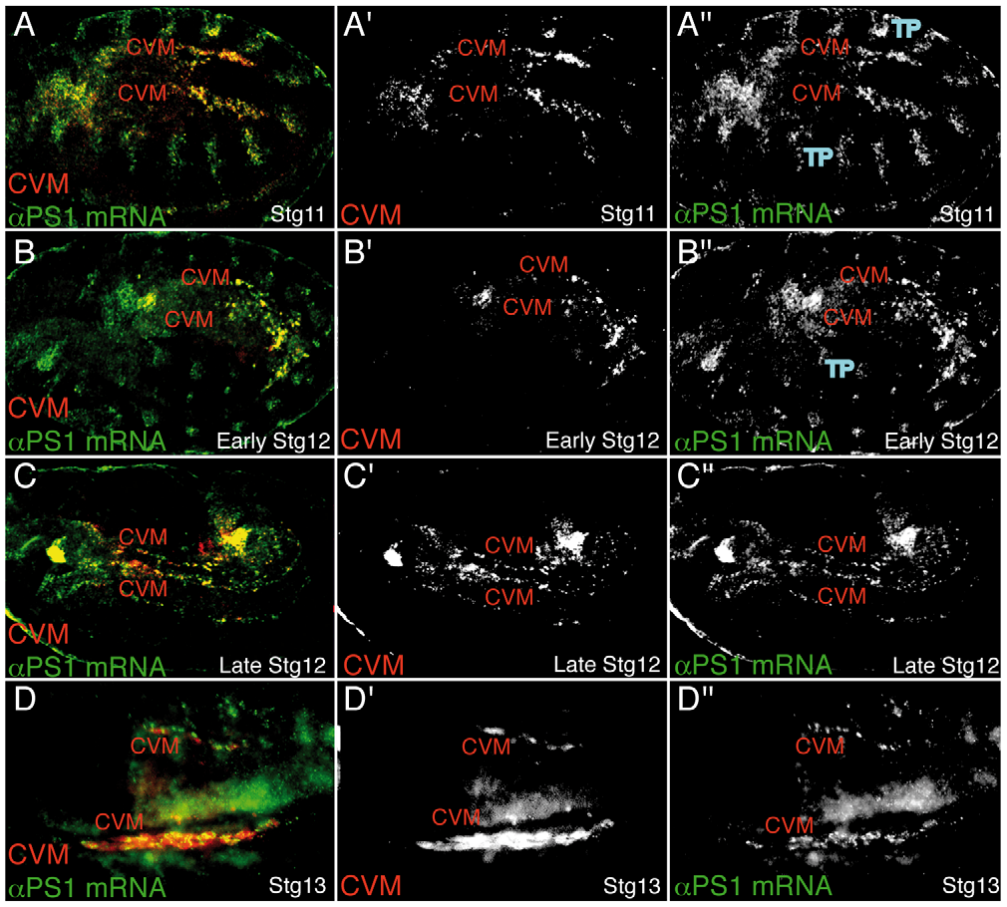
αPS1 is expressed in migrating CVM cells. (A–D) Dorso-lateral views of embryos carrying the CVM marker croc-LacZ double-stained with βGal antibody (red) and αPS1 expression by fluorescence *in situ* hybridization (green). In all panels both clusters of CVM cells (CVM) can be visualized. (A″–D″) αPS1 mRNA is strongly expressed in CVM cells during all phases of their migration, from Stg11 to Stg13.TP, tracheal pits.

CVM cells originate from the posterior tip of the mesoderm anlage. Here, we show that both αPS1 and αPS2 subunits are required for CVM cell migration over the vm. However, previous analysis have shown that while αPS2 is expressed in all mesodermal derivatives, including the vm, αPS1 is only expressed in the embryonic ectoderm and endoderm derivatives [13], [22], [25]. How can we then explain the role of PS1 in CVM migration? To answer this question, we decided to re-examine αPS1 and αPS2 expression patterns during CVM migration. To visualize CVM cells, we used the CVM specific marker *croc-LacZ* [15]. We could detect expression of αPS1 mRNA in CVM cells before germ band retraction, when cells migrate into the space between the germ band and the posterior midgut anlage (Fig. 4A). CVM cells continue to express αPS1 as they separate into two lateral populations and start their migration over the vm (Fig. 4B, C). This expression is maintained through stage 13, when CVM cells become distributed along the trunk region of the embryo (Fig. 4D). The expression persists as the cells begin to surround the midgut and it disappears by stage 14. We could not detect expression of αPS1 in the vm. In contrast, αPS2 was found in the vm, as previously reported, but not in CVM cells (data not shown).

These results show that αPS1 integrin is specifically expressed in cells that migrate over the vm independently of their origin, suggesting that PS1 integrins could have specialized to accomplish the migratory function of integrins. We next decided to test this by analysing the ability of αPS2 to substitute for αPS1 during cell migration.

### Specific requirements for αPS1 function in migrating cells

**Figure 5:**
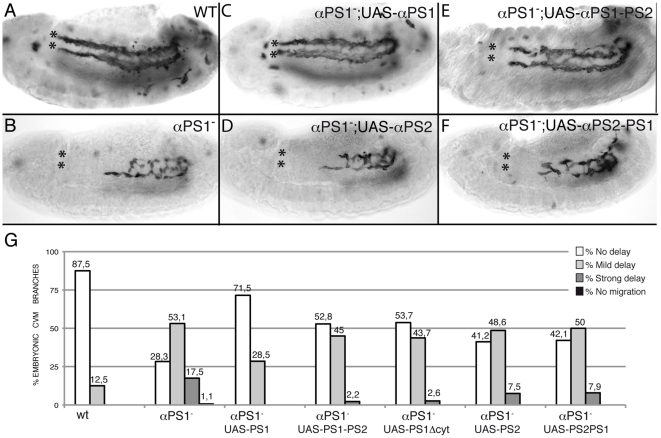
αPS1 function is specifically required in CVM cells to mediate their migration. The ability of different UAS-αPS1 transgenes to rescue the CVM migration defects of Stg 13 αPS1 mutant embryos was assessed by co-expressing UAS-CD2 and the different transgenes using the G447.2-GAL4 and staining with anti-CD2 antibody. (A) Wild type embryo. (B) αPS1 mutant embryo. (C) CVM-specific expression of αPS1, but not αPS2 (D), substantially rescues the CVM migration defects of αPS1 mutant embryos. (E) The αPS1PS2 transgene also shows significant rescue, although less effective than αPS1. (F) Conversely, the αPS2PS1 transgene is as little effective as αPS2. (G) Quantification of the CVM migration phenotype in Stg13 embryos of the indicated genotypes.

The above results suggest a model in which PS1 integrin is required in motile cells for their migration. If this were the case, we should be able to rescue the migration phenotype of *mew* mutant embryos by expressing the αPS1 subunit specifically in the migrating cells. In order to test this, we tried to rescue the CVM migration phenotype of αPS1^−^ mutant embryos by expressing the αPS1 subunit in these cells using the GAL4 line G447.2. We found that the migration defect is substantially rescued, as now 71.5% migrate as wild type and we do not find any branch with a strong delay (Fig. 5C, G). We next tested the specific requirement of PS1 in CVM cells by assessing whether the PS2 integrin could fulfill the role of PS1. Expression of αPS2 in CVM cells is less efficient than αPS1 at rescuing the defects in CVM migration of αPS1 mutant embryos, as in these embryos there is still a proportion of CVM branches, 7.5%, that show a strong delay and only 42.8% migrate normally (Fig. 5D, G). In all cases, we have obtained similar data using at least two independent UAS lines. These results indicate that it is PS1 function in particular, rather than PS integrin function in general, which is required in CVM cells for their migration over the vm.

Previous reports analyzing the specificity of PS integrin function have shown that exchanging the cytoplasmic domains had no effect in the ability of each α subunit to rescue the embryonic lethality due to its own mutation [26]. From these results, it was concluded that the functional differences between the two α subunits lies primarily in the extracellular domains [26]. Here, we have examined the requirement of the αPS1 cytoplasmic tail during CVM migration. We found that although a αPS1 subunit without its cytoplasmic domain, αPS1Δcyt, or with that of αPS2, αPS1–PS2, can substantially rescue the migration defects, it is less efficient than the normal αPS1 subunit (Fig. 5E, G). These results suggest that even though the functional differences between αPS1 and αPS2 lie primarily in their extracellular domains, the cytoplasmic domains may also play a role. Finally, a αPS2 subunit with the αPS1 cytoplasmic tail, αPS2PS1, behaves similar to αPS2 in its inability to rescue the αPS1^−^ mutant phenotype (Fig. 5F, G).

The specificity and requirement of the different domains of αPS1 that we have observed might be particular to CVM migration, due to their mesodermal origin, or they may reflect a general characteristic of αPS1 function during cell migration. In order to test this we repeated the rescue experiments in a population of migratory cells of epithelial origin that also uses the vm as their migratory substrate, the visceral branch (VB) of the embryonic tracheal tree. We had previously shown that αPS1 is also specifically expressed and required in the VB cells for their migration over the vm, as targeted expression of αPS1 in tracheal cells using the breathless-GAL4 (btl-GAL4) line could rescue the visceral branch phenotype of αPS1^−^ mutant embryos [4]. Using the same strategy, we have tested the abilities of the different αPS1 chimeras to fulfill the role of αPS1 in the migrating VB cells, and we have found that they are the same as those here described for the CVM cells (Figure S3).

Thus, we have found similar specific requirements for the different domains of the αPS1 subunit in regulating the migration of cells of two different origins. These results support a model in which αPS1 might have evolved to promote the migratory function of integrins, which relies on a combination of interactions with downstream effectors out and inside the cell.

### PS2 is required in the vm to assemble a proper ECM substrate

**Figure 6:**
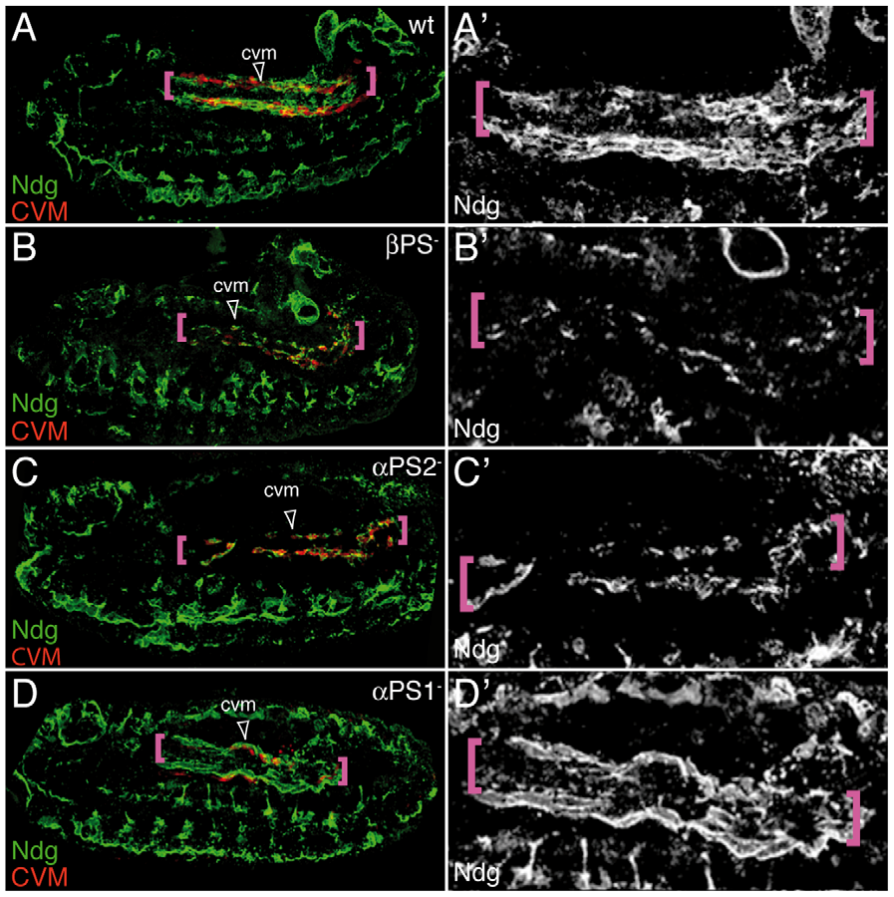
PS2 is required for Nidogen accumulation over the vm. (A–D) Lateral view of Stg13 embryos carrying the CVM marker croc-LacZ double-stained with anti-βGal (red) and anti-Ndg (green) antibodies. (A) In wild type embryos Ndg is found at the edges of vm (pink brackets) along the path of migrating CVM cells. This distribution is affected in βPS (B), αPS2 (C) but not αPS1 (D) mutant embryos.

PS2 has been shown to be required for the migration of different cell populations over the vm [2], [3], [4], [5], [6]. The current model is that PS2 is expressed and required in the vm for these migratory processes. However, the function of PS2 in the vm remains still unknown. PS2 mutant embryos show irregularities in the visceral mesoderm [2], [3]. Therefore, the delay in migration observed in PS2 mutant embryos could be explained as a consequence of irregularities in the substratum. Alternatively, as integrins are required for assembly and/or accumulation of ECM components [27], [28], PS2 could also be required in the vm to assemble an optimal ECM substrate for migration. To test these possibilities, we examined the organization of ECM components over the vm during CVM migration. LamininW (α1,2; β1; γ1) seemed a good candidate as its mRNA was detected in the visceral mesoderm at the time of CVM migration [29], However, we found, in agreement with previous results, that the LamininW protein could not be detected around this tissue until stage 13, when CVM migration has been completed [29]; our own results, data not shown). In contrast, Nidogen (Ndg), another ECM component, was abundantly found closely apposed to the vm from stage 12 onwards (Fig. 6A). Thus, we decided to use Ndg to assess matrix assembly over the vm. We found that in βPS^−^ mutant embryos the accumulation of Ndg was impaired and a spot-like distribution was observed (Fig. 6B). Furthermore, αPS2^−^, but not αPS1^−^, mutant embryos also show an aberrant distribution of Ndg around the vm (Fig. 6C and 6D). These results suggest that an important role for the PS2 integrin in the vm is to assemble a proper ECM substrate required for CVM migration.

Finally, we tested the ability of αPS1 to substitute for αPS2 in the visceral mesoderm. We found that expression of αPS1 in the vm is as efficient as αPS2 at rescuing the CVM migration and Ndg distribution defects of αPS2 mutant embryos (data not shown). These results suggest that both PS1 and PS2 integrins have equivalent abilities in providing a proper ECM substrate for CVM migration.

### The laminin α1,2; β1; γ1 trimer is specifically required for CVM migration

**Figure 7:**
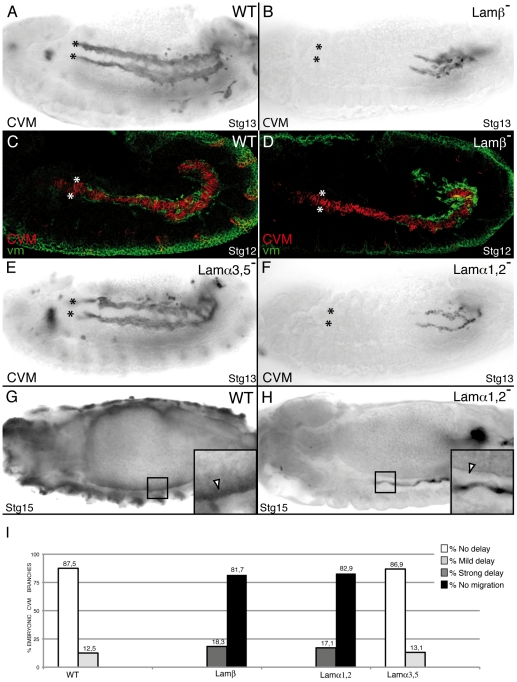
LamininW, but not lamininA, is required for CVM migration. CVM cells are visualized using the combination 5053A-GAL4/UAS-srcGFP (A–D, F, G, H) or G447.2-GAL4/UAS-CD2 (E) and anti-GFP or anti-CD2 antibody staining, respectively. (A) Wild type embryo. (B) In absence of Laminin function, CVM cells fail to migrate. (C) During Stg12, wild type CVM cells (red) migrate in close contact with the vm, visualized with anti-FasIII antibody (green). However, *Lamβ* mutant CVM cells contact an intact vm but fail to migrate (D). (E) CVM migration is unaffected in *Lamα3*, *5* mutant embryos. (F) Conversely, CVM cells of *Lamα1*, *2* mutant embryos show a delay in their migration similar to that observed in *Lamβ* mutant embryos (B). (G, H) Attachment of CVM fibers to the vm is affected in stage 15 *Lamα1*, *2* mutant embryos (H) compare to wild type (G) (arrowhead in magnification in black box). (I) Quantification of the CVM migration phenotype in Stg13 embryos of the indicated genotypes.

As mentioned above, failure of CVM migration in mutant embryos where the vm is abnormal or absent have led to the proposal that the vm serves as a substratum for CVM migration [10], [11]. However, a model has been proposed in which CVM cells do not migrate over the vm directly, but rather they use an ECM substratum present on the vm [12]. This model has not yet been proven and the composition of this putative ECM substratum remains unknown. Here, we have tested this model by analyzing the consequences of eliminating several ECM components, such as laminins, collagen IV and perlecan, on CVM migration. To examine the requirements of laminins during CVM migration, we analyzed embryos mutant for the unique Laminin β chain (Lamβ), which results in the complete elimination of the two laminins present in *Drosophila* [30]. We found that in Lamβ^−^ mutant embryos most CVM cells (81.7%) invaginate but do not move anteriorly (Fig. 7A, B, I). As we have recently shown that the vm of Lamβ^−^ mutant embryos appears highly disorganized at stage 16 [30], the observed disruption of CVM migration in Lamβ^−^ mutant embryos could reflect an earlier requirement for laminins in the vm. However, this does not seem to be the case as no morphological defects were detected in the vm of Lamβ^−^ mutant embryos during CVM migration, stages 10–13 (Fig. 7D). In addition, the normal expression in Lamβ^−^ mutant embryos of reporter genes driven by CVM GAL4 lines strongly suggests that loss of laminin function does not affect CVM specification (Fig. 7B, D). Contrary to vertebrates, in *Drosophila* there are just two laminin trimers: lamininA (α3,5; β1; γ1) and lamininW (α1,2; β1; γ1). To determine whether CVM migration requires either both laminin trimers or a specific trimer, we analyzed CVM migration in embryos mutant for the individual α subunits. We found that while CVM migration was normal in embryos lacking the α3,5 subunit (encoded by *Laminin A*, *LanA*) (Fig. 7E, I), embryos mutant for α1,2 (encoded by *wing blister*, *wb*) showed a CVM migration phenotype that phenocopied the one observed in Lamβ^−^ mutant embryos (Fig. 7F, I). We have recently shown that in absence of all laminins the deposition and assembly of other ECM components, such as Perlecan and Collagen IV, were severely impaired [30]. In this scenario, the role of lamininW during CVM migration could be indirect, whereby lamininW would be required to assemble a proper ECM substrate, or direct, where lamininW is itself required for CVM migration. To test this, we analyzed the distribution of Collagen IV and Perlecan in embryos mutant for Lam α1,2 and found they appeared normal (Figure S4B and data not shown, respectively). In addition, we found that CVM migration was not affected in embryos mutant for Collagen IV, the collagen IV processing factor SPARC or Perlecan (Figure S4D and data not shown). Altogether, these results strongly suggest that lamininW (α1,2; β1; γ1) is one of the main substrates used by CVM cells for their migration. In addition, similarly to integrin mutant embryos, stg15 lamininW mutant embryos also show defects in attachment and spreading of fibers (Fig. 7G, H).

The CVM migration phenotype of embryos lacking laminins is more severe than that found in integrin mutant embryos (compare Fig. 2F with 7B). An explanation for this result could be that CVM cells use other laminin receptors besides integrins to migrate over the vm. Dystroglycan (*Dg*) is another laminin receptor that plays a critical role in linking the ECM and the cytoskeleton. We have analyzed CVM migration in *Dg* mutant embryos and found it was normal (data not shown). In addition, embryos double mutant for integrins and *Dg*, such as αPS1^−^; *Dg^−^* (Figure S4F) or βPS^−^; *Dg^−^* (data not shown), did not show any enhancement of the integrin phenotype. These results show that Laminin function during CVM migration is not mediated by Dg.

### Hemocytes are not required for CVM migration

**Figure 8:**
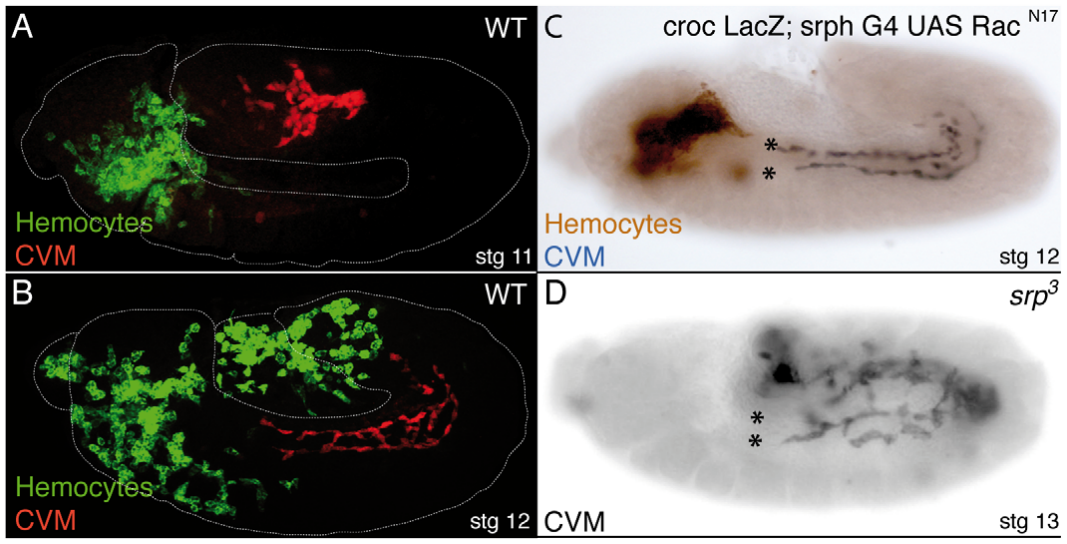
Mature hemocytes and fat body, are not essential for CVM migration. (A–D) Hemocytes and CVM cells are visualized using the lines srph-GAL4/UAS-CD2 and croc-LacZ, respectively. (A) In wild type Stg11 embryos, CVM cells (red) initiate their migration before hemocytes (green) have populated the posterior end of the embryo. (B) In subsequent stages, CVM migration always precedes that of hemocytes. (C) Blocking hemocyte migration (brown), by expressing Rac^N17^, does not affect CVM migration (black). (D) Absence of mature hemocytes and fat body does not affect CVM migration.

The major sources for ECM molecules in the *Drosophila* embryo are hemocytes and the fat body [31]. Hemocytes derive exclusively from the head mesoderm and migrate along several invariant paths throughout the embryo [32]. During late stage 11, hemocytes move anteriorly and ventrally, populating the clypeolabrum and gnathal buds, and posteriorly, beneath the amnioserosa, toward the tail of the embryo [32] and Fig. 8A). As it is around late stage 11 when CVM cells begin to move anteriorly over the vm, they could use the ECM proteins produced by the hemocytes that have populated the end of the germ band. To test this hypothesis, we analyzed how CVM cells move relatively to hemocytes (Fig. 8A, B). We found that CVM migration initiates well before hemocytes have entered the tail end of the embryo (Fig. 8A). Thus, as CVM migration takes place in a hemocyte free region, this suggests that the laminin matrix required for CVM migration might not be laid down by migrating hemocytes. To further test this hypothesis, we blocked completely hemocyte movement by overexpressing a dominant negative form of Rac1, Rac1^N17^, and analyzed the consequences on CVM migration [33]. We found that while hemocytes have not left the head region, CVM cells migrate normally (Fig. 8C). One possible explanation for these results is that hemocytes could produce ECM proteins that are diffused throughout the embryo and maintained over the vm by binding to ECM receptors present in this tissue, such as the PS2 integrins. To test this possibility, we analyzed CVM migration in *serpent (srp)* mutant embryos [34]. In *srp^3^* embryos, the primordium of the hemocytes invaginates with the ventral furrow, but cells fail to proliferate or migrate and subsequently die. As a consequence, *srp^3^* embryos are devoid of mature hemocytes. In addition, although fat body precursors are present, they do not proliferate, do not arrange in a continuous sheet and do not express early differentiation markers such as *seven-up* [34]. Even though *srp^3^* embryos display strong germ band retraction phenotype [35], we found that CVM cells moved anteriorly (Fig. 8D), in contrast to lamininW mutant embryos where CVM cells do not initiate migration (Fig. 7F). These experiments demonstrate that the main sources of ECM molecules, hemocytes and fat body, are not required for CVM migration.

We next decided to test whether the CVM cells and/or the vm could be the source of the ECM substrate by performing RNAi knockdown experiments. RNAi knockdowns of *wb* and *LanB1* via CVM or/and vm Gal4 drivers of the respective UAS-RNAi constructs (see materials and methods) did not affect CVM migration (data not shown). Unfortunately, we believe these experiments are not conclusive, as RNAi does not always work in embryos. In fact, the embryonic CVM cells seem to be particularly refractory to this treatment. Thus, RNAi Knockdown of other genes known to be expressed and required in the CVM, such as HLH54F and *daughterless*, has been shown to work in the adult CVM cells but not in the embryo [16].

## Discussion

Integrin cell surface adhesion receptors play an essential role in mediating cell migration during development. During *Drosophila* embryogenesis, integrins are implicated in the movement of three groups of epithelial cells over the vm [3], [4], [6]. In all cases, migration requires the function of PS1, and in some cases PS3, in migrating cells and an unknown function for PS2 in the visceral mesoderm. Here, we show that PS1 and PS2 integrins are also required for the migration over the vm of cells of mesodermal origin, the longitudinal visceral muscle progenitors or CVM. We show that PS1 is expressed and required in migrating CVM cells and that this function is highly specific, as it cannot be replaced by PS2. Furthermore, we demonstrate that this specificity lies mainly in the extracellular domain. In addition, our results revealed that a function of PS2 in the vm is to assemble an ECM substrate necessary for migration. This function of PS2 is not specific, as it can be substituted by PS1. Finally, we identify the trimer lamininW (α1,2; β1; γ1) as the main ECM component supporting CVM migration.

Previous analysis of the capacities of PS1 and PS2 integrins to substitute for each other in several cellular processes have revealed specific requirements for the different functional abilities of the two integrins [2], [3], [4], [26], [36], [37]. In a classical view, the diversification of the function of a progenitor gene may occur through the partitioning of regulatory sequences in the original duplication event. The differential tissue expression would then lead to functional refinement and diversification [38]. Phylogenetic analyses of α integrins support this model for the PS1 and PS2 families [39]. An ancestral single α subunit would have duplicated to give rise to αPS2, which retained mesodermal regulatory sequences and evolved to perform stable adhesions, and αPS1, which retained ectodermal and endodermal regulatory elements, and became specialized in mediating dynamic adhesions [26]. Our results showing that αPS1 is expressed in a mesodermal derivative suggest that the scenario might not be that simple. In an alternative model, protein functional divergence may have preceded changes in expression patterns [40]. After the duplication event, rapid changes in functionally important sequences of the protein may have gradually led to functional divergence. Then, the two duplicate genes might have undergone degeneration of some of their cis-regulatory motifs. In this context, αPS1 would have diverged to perform a migratory function. Finally, coding-sequence and expression divergence between αPS2 and αPS1 could have been coupled.

We show here that during cell migration, as it is the case for most integrin-dependent developmental events, PS1 and PS2 cannot substitute for each other [3], [4], [26], [41]. Our results demonstrate that the specificity resides mainly in the extracellular domain. PS1 and PS2 integrins show distinct binding specificity for all ligands identified to date. Thus, one way to explain why the αPS2 extracellular domain cannot substitute the αPS1 could be that the interaction between PS2 and its own ligand might promote an adhesion that it is not appropriate for cell migration. The cytoplasmic domain of the α subunits plays important roles in specifying responses upon ligand-binding. For example, while α5 and α2 cytoplasmic domains support collagen gel contraction, α4 cytoplasmic domain promotes cell migration on collagen [42]. Our demonstration that the αPS2 cytoplasmic tail cannot fully replace the αPS1 tail suggests that during cell migration the cytoplasmic domains may also participate by transmitting distinct intracellular responses. Alternatively, the downstream effectors of PS2 may not be present in migrating cells. Finally, as PS2 has been shown to promote cell spreading on tiggrin [43], a third explanation could be that the ECM components that serve as PS2 ligands for migration are not present on the vm.

In contrast, we show here that αPS2 can be replaced by αPS1 in the vm, as it is the case during the migration of the tracheal cells [4]. This result suggests that PS integrin function during the assembly of an ECM substrate necessary for cell migration does not require specificity of the α subunit. This is also the case for integrin function on the regulation of gene expression in the embryo [37].

During amphibian gastrulation, integrins contribute to cell movement in several ways. They are required in migrating cells to provide traction and to transmit guidance signals and in the surrounding cells to assemble an ECM substrate that serves as tracks for migration [44], [45]. In *Drosophila*, integrins have been shown to be required for the assembly of ECM components in several developmental processes, including dorsal closure, wing imaginal disc morphogenesis and maintenance of the stem-cell niche in the gonads. Here, we show that in flies, as it is the case in vertebrates, integrins, and in particular PS2, contribute to cell migration by assisting in the assembly of an ECM substrate. PS2, and not PS1, mutant embryos show irregularities in the visceral mesoderm. Thus, there are at least two possible ways in which PS2 could function in the assembly of an ECM over the vm. PS2 could be required for proper morphogenesis of the vm, which in turn could be necessary for proper expression and/or localization of ECM components. Alternatively, PS2 could directly assemble an ECM essential for both cell migration and proper vm morphogenesis.

Experiments in different model systems have demonstrated the importance of the ECM for the migration of different cell populations [46]. We recently showed that removal of all laminins in the *Drosophila* embryo affects the migration of several cell populations over the vm [30]. Here, we show that laminins are also required for the migration of CVM cells. Furthermore, our results demonstrate that lamininW is the main ECM molecule supporting CVM migration, and that lamininA, perlecan and collagen IV appear of lesser importance. The migration defects observed in embryos mutant for lamininW are more severe than those found in embryos lacking both the PS integrins and Dg. One explanation for this result is that other laminin receptors might be required for CVM migration. Alternatively, laminin function during CVM migration may not only be to provide an ECM substratum, but also to present binding sites for guidance cues necessary for CVM migration. Recent studies in *Drosophila* showing genetic interactions between laminins and the secreted guidance cue Slit during embryonic cardiac cell migration and axons across the midline support this option [47], [48]. We have also found that Ndg, a glycoprotein that forms a non-covalent complex with laminin and collagen IV, accumulates over the vm at the time of CVM migration. Besides a structural role in the generation and maintenance of basement membranes, Ndg, free of laminins, has its own biological functions that include cell migration. Ndg is required for the migration of trophoblasts cells, neutrophils and Schwann cells [49], [50]. Interestingly, α3β1 integrins, which is most similar to αPS1βPS, seem to mediate this non-structural function of Ndg [51]. A role for *Drosophila* Ndg in cell migration awaits the isolation of mutants in this gene. Finally, we show that the main source of ECM molecules in *Drosophila*, hemocytes and fat body [52], [53], do not provide the ECM molecules required for CVM migration, suggesting that there might be an alternative source. The alternative source could be either the vm itself, as lamininW has been detected in this tissue in stage 11 embryos [29], [54], or the CVM themselves. This is the case for human keratinocytes, which deposit laminin 332 to promote their linear migration [55].

During embryonic development, many cells use as their migratory routes ECM components found in the interstices and/or basement membrane surrounding different tissues. The interactions between moving cells and ECM components has to be highly coordinated to guide cells towards their final destinations during the process of organogenesis. The understanding of how this is regulated is still rather fragmentary due to the complexity of the cellular and molecular interactions involved. We have recently shown that in the *Drosophila* embryo, ECM components, such as laminins, are required for the migration of many cell populations, including endoderm, macrophages, salivary glands, trachea and mesodermal cells [30] and this work). Thus, we can use the power of *Drosophila* as a model system to gain insights into the developmental roles of the cell-ECM interactions during cell migration. In addition, migrating tumour cells destroy and consequently rearrange the ECM that surrounds them in order to promote proliferation and tumour invasion. Thus, a detailed and comprehensive analysis of the role of the different ECM molecules and their regulation during cell migration is important to further understand not only embryonic development but also tumor metastasis.

## Materials and Methods

### *Drosophila* strains and techniques

The following stocks were used: *βν* [5], *bHLH54F-LacZ* and *DocF4s1 GFP* [16], *biniou* (Bloomington), *croc*-LacZ [15], *Dg^323^* [56], *if^B4^* [24], *LanA^9–32^* [57], *Lamβ^DEF^* [30], *mew^m6^* [21], *mys^XG43^* FRT101 [58], *rhea^79^* FRT 2A [59], *scab^IIG^* [23], *srp^3^* [34], *trol^null^* [60], *wb^SF11^* [29], *ovo^D1^FRT101*, *ovo^D1^FRT2A*, *Df(2L)BSC172*, FM7eve-LacZ, FM7twi-*GFP*, CyO*slp*-LacZ and TM3*ftz*-LacZ (Bloomington). For missexpression experiments, we used the GAL4/UAS system [61]. The following lines were used: G447.2 [9], 5053A GAL4 [14], btl GAL4 [62], srph-GAL4 (a gift from R. Reuter), UAS-CD2 [63], UAS src-GFP and UAS-TauGFP (Bloomington). For the rescue experiments we used the following UAS lines: UAS-αPS1^3.4,3.5^, UAS-αPS2^3A,3B^, UAS-αPS1PS2^2.1,3.1^, UAS-αPS2PS1^2A,2B^ [26] and UAS-αPS1ΔCyt^1,2^ [64]. For the RNAi knockdown experiments, we have used the following UAS-RNAi lines, UAS-RNAi LanB1 (VDRC 23119 and 23121), UAS-RNAi wb (VDRC 101561 and 1560), and yv;PTRiP JF03238 for *wb*, and combinations of GAL4 lines in the CVM and in the vm: G447Gal4;5053Gal4 and HLH5FdGal4;5053Gal4 for the CVM and bap3G4;24BGal4, twi-Gal4;24BGal4 for the vm. In all cases, UAS-dicer2 was co-expressed to enhance the silencing effect.

### Histochemistry

Whole-mount *in situ* hybridization and antibody staining of embryos were performed using standard procedures. We used the following primary antibodies: Rb-βGal 1/2000 (Cappel Lab), Mo-βGal 1/10000 (Promega), Mo-CD2 1/200 (OX-34, Serotec), GP-ColIV 1/500 (a gift from Ringuette M), Rb-Couch potato 1/200 (a gift from Bellen H), Mo-Fas III 1/4 (7G10, Developmental Studies Hybridoma Bank), Mo-GFP 1/300 and Rb-GFP 1/100 (Molecular Probes), Rb-Perlecan 1/2000 [65], Rb-Ndg 1/100 [66]. Alexa-conjugated secondary antibodies used were Alexa 488 (green), Alexa 568 (red) (Molecular Probes™). For non-fluorescent staining, embryos were incubated in biotinylated secondary antibodies followed by incubation with Elite ABC complex (Vector Laboratories) and revealed with DAB (Gibco-BRL). For fluorescent *in situ* hybridization, the Streptoavidin-fluorescein was used in combination with the Tyramide Signal Amplification kit (NEL700A, Perkin-Elmer). Images were collected with a Zeiss Axioplan 2 microscope or a Leica TCS-SP2 confocal microscope.

### Generation of germline clones and rescue experiments

To generate *mys*, *mys;βν* and *rhea* germline clones, the FLP-recombinase system was used [67]. Virgin females of the genotype *yw mys^XG43^* FRT101, *yw mys^XG43^* FRT101; *βν* or *yw* hs flp^1.22^; *rhea^79^* FRT2A were crossed with males *w ovo^D1^* FRT101; hsFLP38, *w hsflp ovo^D1^* FRT101; *βν* and *ovo^D1^* FRT2A respectively. Larvae of these crosses were heat-shocked at 37°C for 2 hours. The female progeny *yw mys^XG43^* FRT101*/w ovo^D1^* FRT101; hsFLPF38*/+*, *yw mys^XG43^* FRT101*/w hsflp ovo^D1^* FRT101; *βν* and *yw* hs flp 1.22/+; *rhea^79^* FRT2A/*ovo^D1^* FRT2A were then crossed to males FM7*eve*LacZ; G447-UAS-CD2, FM7*eve*LacZ; *βν*; 5053A-UAS-srcGFP or G447.2GAL4-UAS-CD2; *rhea^79^*FRT2A/TM3*ftz*LacZ respectively.

To compare rescue abilities of the different transgenes, a mean out of an average of 35 embryos per genotype was calculated. These mean values were ranked transformed and analysed with the analysis of variance (ANOVA). Differences among genotypes were tested using post-hoc Fisher (LSD) [68].

### *In vivo* time-lapse recording

Embryos were collected, dechorionated and mounted on a glass coverslip. They were immersed in Voltalef oil 10S and the coverslip was attached to a steel frameslide (no. 11505151; Leica, Wetzlar, Germany). GFP-fluorescent images were captured using a Leica TCS-SP2 confocal microscope. Movies were assembled from single focal plane images using ImageJ.

## Supporting Information

Figure S1 **CVM migration in** ***biniou*** **and** ***talin*** **mutant embryos.** (A–D) CVM from Stg13 embryos are visualized using the combination G447.2GAL4/UAS-CD2. (A) Wild type. (B) CVM cells in *biniou* mutant embryos migrate up to the level of A4. (C, D) CVM from *talin* maternal and zygotic mutant embryos (D) phenocopies the migration defects observed in βPS maternal and zygotic mutant embryos (C). Note that both βPS and *talin* mutant embryos also display a germ band retraction phenotype.
(TIF)

Figure S2 **Loss of integrin function does not affect CVM specification.** (A, C, E and G) wt embryos and (B, D, F and H) βPS maternal and zygotic mutant embryos. CVM from Stg 13 embryos are visualized using different CVM markers: bHLH54F-LacZ (A, B), Doc-GFP (C, D), anti-Couch-potatoe (Cpo) antibody (E, F) and croc-LacZ (G, H). These markers are normally expressed in βPS mutant CVM cells (B, D, F and H).
(TIF)

Figure S3 **αPS1 function in tracheal cells during their migration is highly specific.** The ability of different UAS-αPS1 transgenes to rescue the tracheal visceral branch migration defects of αPS1 mutant embryos have been tested by co-expressing UAS-tauGFP and the different transgenes using btl-GAL4 and staining with anti-GFP antibody. Rescue was tested at Stg14 when wild type tracheal visceral branches have completed their migration reaching the foregut-midgut transition (*). (A) Wild type embryo. (B) αPS1 mutant embryo. (C) Tracheal expression of αPS1PS2 rescues substantially, although less efficiently than αPS1. (D) Conversely, a αPS2PS1 transgene is as little effective as αPS2. (E) Quantification of the tracheal visceral branch migration phenotype of the indicated genotypes.
(TIF)

Figure S4 **Collagen IV and Dystroglycan are not required for CVM migration.** (A, B) Stage 14 embryos stained with an anti- Collagen IV (Col IV) antibody. (A) Col IV localization in the ventral nerve cord (arrowhead) and around the midgut (arrow) is lost in Lamβ embryos, but not in Lamα1,2 mutant embryos (B). (C, D) *Df(2L)BSC172*/CyOslp-LacZ; 5053A-GAL4/UAS-srcGFP Stg13 embryos stained with anti-GFP (red) and anti-βgal (green) antibodies to visualize CVM cells and the balancer, respectively. CVM migration is not affected in Col IV mutant embryos (D). (E, F) Stg13 embryos stained with an anti- Couch-potatoe (Cpo) antibody. Elimination of Dg did not enhance the CVM migration defects (arrowhead) from αPS1 mutant embryos.
(TIF)

Movie S1 **In vivo migration of CVM cells.** Time-lapse movie of wild type CVM cells expressing src-GFP. During the process of germ band retraction, CVM cells migrate anteriorly. * and + indicate the original and final positions of CVM cells, respectively.
(MOV)

Movie S2 **CVM cells send projections while migrating.** Time-lapse movie of wild type CVM cells expressing src-GFP. Cell protrusions (white arrowhead) can be observed as cells migrate.
(MOV)

Movie S3 **βPS mutant CVM cells can form cellular protrusions while migrating.** Time-lapse movie of βPS mutant CVM cells expressing src-GFP shows how elimination of βPS function does not impair the formation of cellular protrusions (white arrowhead).
(MOV)
