# Supplementary figures and images for: PS Integrins and Laminins: Key Regulators of Cell Migration during Drosophila Embryogenesis
Source: PLoS One. 2011 Sep 16;6(9):e23893. doi: 10.1371/journal.pone.0023893 (PMC3174947; doi:10.1371/journal.pone.0023893)

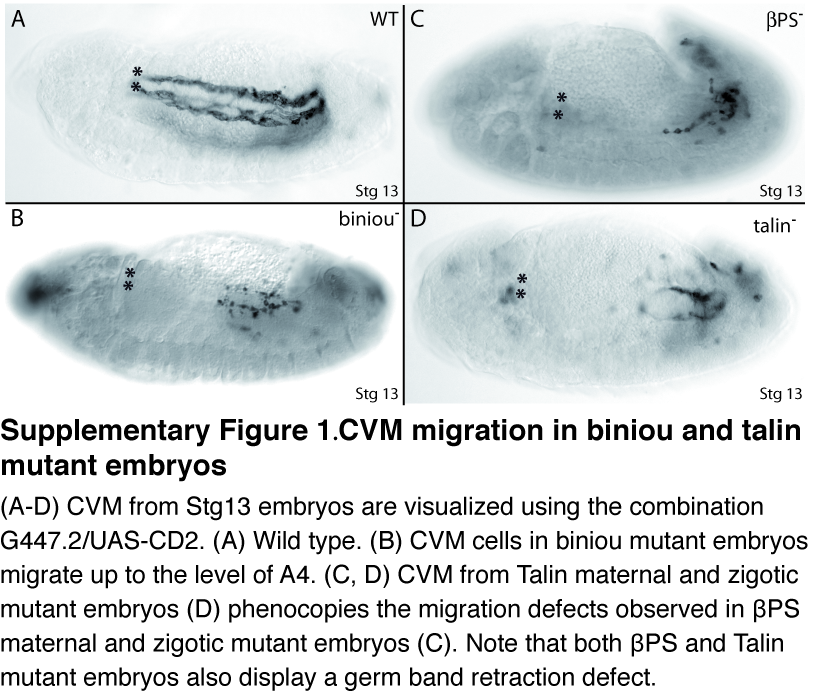

Supplement: Figure S1 — CVM migration in biniou and talin mutant embryos. (A–D) CVM from Stg13 embryos are visualized using the combination G447.2GAL4/UAS-CD2. (A) Wild type. (B) CVM cells in biniou mutant embryos migrate up to the level of A4. (C, D) CVM from talin maternal and zygotic mutant embryos (D) phenocopies the migration defects observed in βPS maternal and zygotic mutant embryos (C). Note that both βPS and talin mutant embryos also display a germ band retraction phenotype. (TIF) [file pone.0023893.s001.tif]

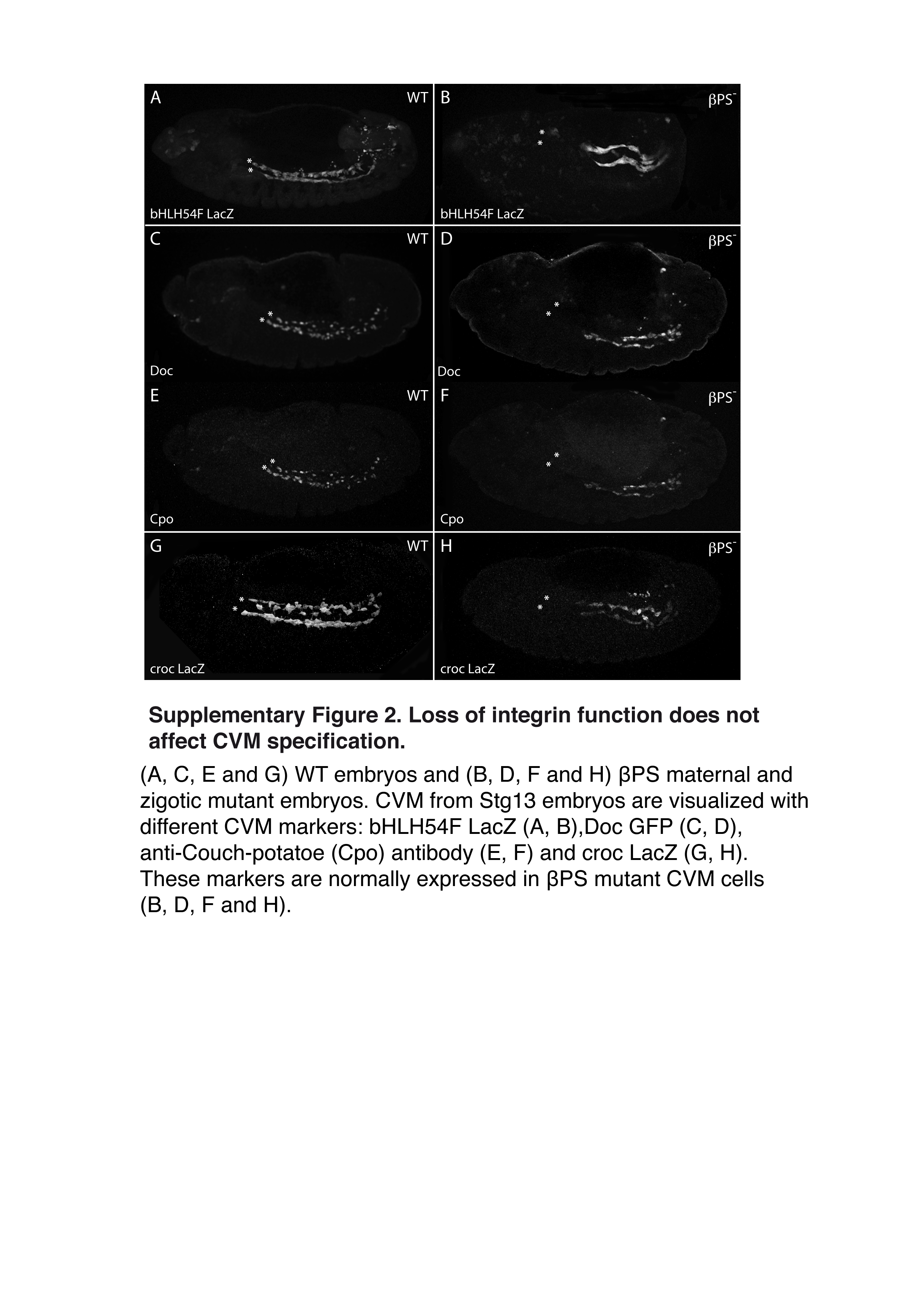

Supplement: Figure S2 — Loss of integrin function does not affect CVM specification. (A, C, E and G) wt embryos and (B, D, F and H) βPS maternal and zygotic mutant embryos. CVM from Stg 13 embryos are visualized using different CVM markers: bHLH54F-LacZ (A, B), Doc-GFP (C, D), anti-Couch-potatoe (Cpo) antibody (E, F) and croc-LacZ (G, H). These markers are normally expressed in βPS mutant CVM cells (B, D, F and H). (TIF) [file pone.0023893.s002.tif]

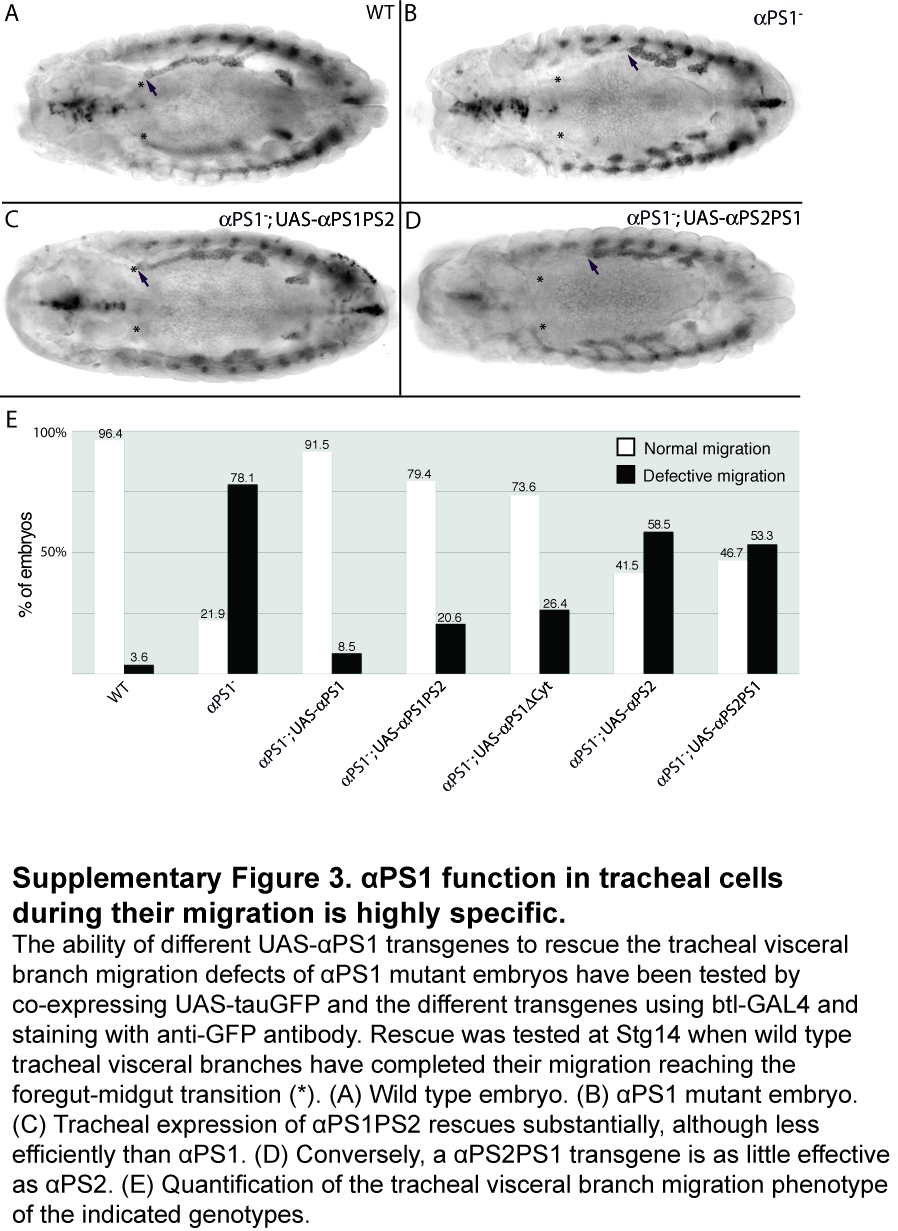

Supplement: Figure S3 — αPS1 function in tracheal cells during their migration is highly specific. The ability of different UAS-αPS1 transgenes to rescue the tracheal visceral branch migration defects of αPS1 mutant embryos have been tested by co-expressing UAS-tauGFP and the different transgenes using btl-GAL4 and staining with anti-GFP antibody. Rescue was tested at Stg14 when wild type tracheal visceral branches have completed their migration reaching the foregut-midgut transition (*). (A) Wild type embryo. (B) αPS1 mutant embryo. (C) Tracheal expression of αPS1PS2 rescues substantially, although less efficiently than αPS1. (D) Conversely, a αPS2PS1 transgene is as little effective as αPS2. (E) Quantification of the tracheal visceral branch migration phenotype of the indicated genotypes. (TIF) [file pone.0023893.s003.tif]

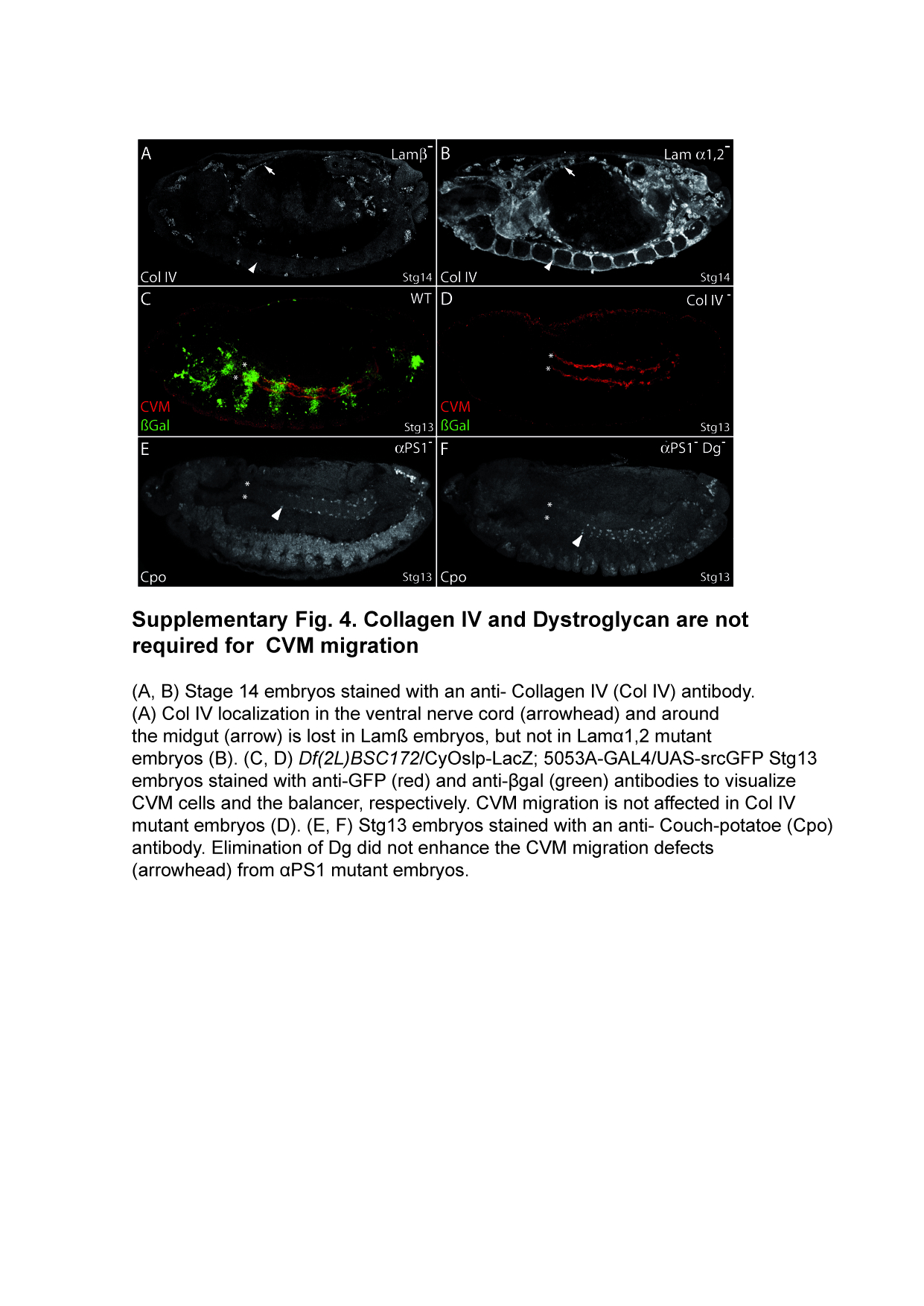

Supplement: Figure S4 — Collagen IV and Dystroglycan are not required for CVM migration. (A, B) Stage 14 embryos stained with an anti- Collagen IV (Col IV) antibody. (A) Col IV localization in the ventral nerve cord (arrowhead) and around the midgut (arrow) is lost in Lamβ embryos, but not in Lamα1,2 mutant embryos (B). (C, D) Df(2L)BSC172/CyOslp-LacZ; 5053A-GAL4/UAS-srcGFP Stg13 embryos stained with anti-GFP (red) and anti-βgal (green) antibodies to visualize CVM cells and the balancer, respectively. CVM migration is not affected in Col IV mutant embryos (D). (E, F) Stg13 embryos stained with an anti- Couch-potatoe (Cpo) antibody. Elimination of Dg did not enhance the CVM migration defects (arrowhead) from αPS1 mutant embryos. (TIF) [file pone.0023893.s004.tif]
